# Cyclin D1 Binding Protein 1 Responds to DNA Damage through the ATM–CHK2 Pathway

**DOI:** 10.3390/jcm11030851

**Published:** 2022-02-06

**Authors:** Yusuke Niwa, Kenya Kamimura, Kohei Ogawa, Chiyumi Oda, Yuto Tanaka, Ryoko Horigome, Masato Ohtsuka, Hiromi Miura, Koichi Fujisawa, Naoki Yamamoto, Taro Takami, Shujiro Okuda, Masayoshi Ko, Takashi Owaki, Atsushi Kimura, Osamu Shibata, Shinichi Morita, Norihiro Sakai, Hiroyuki Abe, Takeshi Yokoo, Akira Sakamaki, Hiroteru Kamimura, Shuji Terai

**Affiliations:** 1 Division of Gastroenterology and Hepatology, Graduate School of Medical and Dental Sciences, Niigata University, Niigata 951-8510, Niigata, Japan; yusuke.28chan@gmail.com (Y.N.); kogawa@med.niigata-u.ac.jp (K.O.); chiyumio@med.niigata-u.ac.jp (C.O.); ytanaka@med.niigata-u.ac.jp (Y.T.); hrgm_ryonryon1127@nifty.com (R.H.); mkou@med.niigata-u.ac.jp (M.K.); towaki@med.niigata-u.ac.jp (T.O.); yo-atsu@castle.ocn.ne.jp (A.K.); oshibatai@med.niigata-u.ac.jp (O.S.); s-morita@med.niigata-u.ac.jp (S.M.); nsakai@med.niigata-u.ac.jp (N.S.); hiroyukiabe@med.niigata-u.ac.jp (H.A.); t-yokoo@med.niigata-u.ac.jp (T.Y.); saka-a@med.niigata-u.ac.jp (A.S.); hiroteruk@med.niigata-u.ac.jp (H.K.); terais@med.niigata-u.ac.jp (S.T.); 2 Department of General Medicine, Niigata University School of Medicine, Niigata 951-8510, Niigata, Japan; 3 Department of Molecular Life Science, Division of Basic Medical Science and Molecular Medicine, School of Medicine, Tokai University, Isehara 259-1193, Kanagawa, Japan; om29859@tsc.u-tokai.ac.jp (M.O.); rascal511520531@yahoo.co.jp (H.M.); 4 Department of Gastroenterology and Hepatology, Yamaguchi University Graduate School of Medicine, Ube 755-8505, Yamaguchi, Japan; fujisawa@yamaguchi-u.ac.jp (K.F.); nao-yama@yamaguchi-u.ac.jp (N.Y.); t-takami@yamaguchi-u.ac.jp (T.T.); 5 Division of Bioinformatics, Graduate School of Medical and Dental Sciences, Niigata University, Niigata 951-8510, Niigata, Japan; okd@med.niigata-u.ac.jp

**Keywords:** Ccndbp1, Atm, Ezh2, Chk2, DNA damage, hepatocellular carcinoma

## Abstract

Cyclin D1 binding protein 1 (CCNDBP1) is considered a tumor suppressor, and when expressed in tumor cells, CCNDBP1 can contribute to the viability of cancer cells by rescuing these cells from chemotherapy-induced DNA damage. Therefore, this study focused on investigating the function of CCNDBP1, which is directly related to the survival of cancer cells by escaping DNA damage and chemoresistance. Hepatocellular carcinoma (HCC) cells and tissues obtained from *Ccndbp1* knockout mice were used for the in vitro and in vivo examination of the molecular mechanisms of CCNDBP1 associated with the recovery of cells from DNA damage. Subsequently, gene and protein expression changes associated with the upregulation, downregulation, and irradiation of CCNDBP1 were assessed. The overexpression of *CCNDBP1* in HCC cells stimulated cell growth and showed resistance to X-ray-induced DNA damage. Gene expression analysis of *CCNDBP1*-overexpressed cells and *Ccndbp1* knockout mice revealed that Ccndbp1 activated the Atm–Chk2 pathway through the inhibition of *Ezh2* expression, accounting for resistance to DNA damage. Our study demonstrated that by inhibiting *EZH2*, *CCNDBP1* contributed to the activation of the ATM–CHK2 pathway to alleviate DNA damage, leading to chemoresistance.

## 1. Introduction

Cyclin D1 binding protein 1 (CCNDBP1, also known as GCIP and HHM) is a cyclin D-binding dominant-negative helix–loop–helix protein with no DNA binding region [1,2]. It is expressed in various tissues, including the thymus, spleen, liver, small intestine, colon, brain, muscle, heart, kidney, lung, and peripheral leukocytes [3,4]. Furthermore, it interacts with various proteins, including cyclin D1 [5], SYF2 [6,7,8], E12 [4], CT847 [9], Jab1 [10], Sirt6 [5], MyoD [11], and Olig1 [12]. The molecular mechanisms of the interaction of CCNDBP1 with these proteins involve G1/S cell cycle phase progression in hepatocytes [13]; tumor suppression in liver cancer [10,14,15,16], breast cancer [15,17,18,19], gastric cancer (GC) [19], lung cancer [20], prostate cancer [19], and colon cancer [5]; regulation of *TGF-β* target genes, including the *Olig1-Smad* synexpression group [12]; and regulation of skeletal myogenesis interacting with *MyoD* [11]. The significant downregulation of *CCNDBP1* in non-immortalized human cell lines was recently reported to result in chromosomal imbalance [21]. In addition, Takami et al. reported that CCNDBP1 protein expression in hepatocellular carcinoma (HCC) was associated with pathologic differentiation by demonstrating its expression upon the initiation of hepatocarcinogenesis and a more positive staining in well-differentiated HCC than in poorly differentiated HCC [10]. These results indicated the possibility that CCNDBP1 is expressed upon cell proliferation in the early stage of malignant transformation [22] and contributes to the maintenance of chromosomal stability as a tumor suppressor of malignant cell growth; suppression of its function may further enhance the malignant potential. The similar mechanism of tumor suppressor gene, *Bcl11b*, has been reported by showing the vulnerability of cells and tissues to DNA replication stress and damages. These reports implicate these tumor suppressor genes in the remedy for DNA replication stress and maintenance of genomic integrity [23,24].

Furthermore, *CCNDBP1* methylation in cancer cells was reported to be related with the chemosensitivity of colorectal cancer to 5-fluorouracil [25], suggesting that *CCNDBP1* expression in cancer cells might be related with tumor viability upon the initiation of antitumor agents. These results were supported by evidence that its interactor SYF2 induced apoptosis in neuronal [6] and retinal ganglion cells [8]; however, upregulation of SYF2 in esophageal cancer cells contributed to the chemoresistance and caused DNA damage in the cells. These results indicated that when expressed in tumor cells, *CCNDBP1* may contribute to the viability of cancer cells by rescuing them from DNA damage [7].

*CCNDBP1* functions as a tumor suppressor as it negatively regulates TGF-β signal-induced cell migration depending on the surrounding condition [26]. The direct interaction of MEK2 was recently reported to phosphorylate CCNDBP1 at its Ser313 and Ser356 residues, thereby promoting its turnover by ubiquitin-mediated proteasomal degradation, which led to cancer cell proliferation, migration, and invasion [27]. However, the function of CCNDBP1 that is directly related with the survival from DNA damage and chemoresistance in cancer cells has not been investigated. Therefore, this study aimed to investigate the molecular mechanisms of *CCNDBP1*, focusing on the recovery from DNA damage of liver cancer cells and in *Ccndbp1* knockout mice.

## 2. Materials and Methods

### 2.1. Plasmids

The CCNDBP1-expressing plasmid, which contained a chicken β-actin promoter, cytomegalovirus enhancer, and an IRES, was generated through a multistep and ligation-based cloning procedure using the full-length complementary DNA of the human homolog of Ccndbp1. The plasmid was purified using a Plasmid Mega Kit (Qiagen, Hilde, Germany). The purity of the plasmid preparation was checked by absorbance at 260 and 280 nm and 1% agarose gel electrophoresis.

### 2.2. Cells

Human hepatoma HLE and HepG2 cell lines were purchased from the Japanese Collection of Research Bioresources Cell Bank (National Institutes of Biomedical Innovation, Health and Nutrition, Ibaraki, Osaka) and were cultured in Dulbecco’s Modified Eagle Medium (gibco, 11885-084, Thermo Fisher Scientific, Waltham, MA, USA), which contained 10% fetal bovine serum and 100 IU/mL of penicillin and streptomycin. Cells were placed in a 5% CO_2_-humidified incubator at 37 °C. Either mock or *CCNDBP1* cloned vectors were transfected into the HLE and HepG2 cells using FuGENE HD Transfection Reagent (Promega, Madison, WI, USA), followed by G418 sulfate selection. From each of the four cell lines, three independent clones were isolated and used for assay.

The expressions of *CCNDBP1* and glyceraldehyde 3 phosphate dehydrogenase (*GAPDH*) genes, and CCNDBP1 and β-actin proteins in the cell lines were confirmed by reverse transcription polymerase chain reaction (PCR) or Western blotting. For these analyses, the RNA Easy Mini kit (Qiagen, Valencia, CA) was used to prepare the total RNA from cells, according to the protocol recommended by the manufacturer. Using SuperScript II Reverse Transcriptase (Invitrogen, Carlsbad, CA, USA), complementary DNA was synthesized from 1–5 mg of total RNA using an oligo (dT) primer; 1–2 aliquots of complementary DNA products PCR were used with the following primers:

CCNDBP1 (forward): GCTGTGGAAGAATGTGACC
CCNDBP1 (reverse): AGAGCCAAATCATCCACA
GAPDH (forward): AGGTCGGTGTGAACGGATTTG
GAPDH (reverse): TGTAGACCATGTAGTTGAGGTCA

Multiplex PCR was carried out similarly, and GAPDH primers were always included as the reference. The PCR products were separated by electrophoresis in 1% agarose gel and stained with ethidium bromide for visualization. The PCR protocol was as follows: 10 min at 95 °C, followed by 35 cycles (30 s at 95 °C, 30 s at 55 °C and 1 min at 72 °C) and a 7-min extension at 72 °C. The details of the Western blotting are summarized in Section 2.6.

### 2.3. Animals

All animal experiments were approved by and conducted in full compliance with the regulations of the Institutional Animal Care and Use Committee at Niigata University, Niigata, Japan. Male BALB/c mice (*n* = 50, 8 weeks of age, 25–30 g) were purchased from CLEA Japan, Inc. (Tokyo, Japan). *Ccndbp1* knockout mice (*n* = 50, 8 weeks of age, and 25–30 g) were kindly provided by Yamaguchi University. The mice were housed in specific pathogen-free facilities under standard conditions at a temperature of 20–23 °C and humidity of 45–55% and were fed a standard diet.

### 2.4. Irradiation

The cells and mice were irradiated with X-ray at 160.0 kV, 5.0 mA, 400 mm, and 0.8 Gy/min for 3 minutes and at 160.0 kV, 5.0 mA, 400 mm, and 1 Gy/min for 10 min using MBR-1605RA (Hitachi Power Solutions Co., Ltd., Hitachi, Ibaraki, Japan). The samples from the cells were collected at appropriate time points, and animals were sacrificed at 12 h after irradiation for tissue collection according to the previous reports [23,24] and preliminary results.

### 2.5. Cell Growth Assay

Mock-HLE, Mock-HepG2, CCNDBP1-HLE, and CCNDBP1-HepG2 cells were plated in 96-well tissue culture dishes at 1 × 10^4^ cells per well in 100 μL of the aforementioned medium and were treated with and without X-ray (0.8 Gy/min, 3 min) or 20 µM of cisplatin (CDDP). The reagent 3-(4,5-dimethylthiazol-2-yl)-2,5-diphenyltetrazolium bromide (MTT) was added to the cells at the indicated times after treatment, followed by counting with Premix WST-1 Cell Proliferation Assay System (Takara Inc., Kyoto, Japan), according to the instructions supplied.

### 2.6. Western Blotting

The cells and tissues samples from the mice were collected for Western blotting at the appropriate time points; suspended in phosphate-buffered saline; and mixed with an equal volume of lysis buffer, 50-mM Tris–HCl (pH 8.0), 1.5% TritonX-100, 150-mM NaCl, 1-mM CaCl2, 1-mM MgCl2, and protease inhibitor; and homogenized with an electric homogenizer. The extract was subjected to gel electrophoresis using 8–16% MINI-PROTEAN TGX Stain Free Gels (No. 456-8105; Bio-Rad Laboratories, Inc., Hercules, CA, USA) and blotted onto PVDF membranes (Transfer Pack, No. 1704156; Bio-Rad Laboratories, Inc., Hercules, CA, USA) by Trans-Blot Turbo (No. 1704150J1; Bio-Rad Laboratories, Inc., Hercules, CA, USA) using either 10× Tris/ Glycine/ SDS buffer (No. 161-0732; Bio-Rad Laboratories, Inc., Hercules, CA, USA) or 10× Tris/ CAPS Buffer (No. 161-0778; Bio-Rad Laboratories, Inc., Hercules, CA, USA). The membranes were then blocked by EzBlockChemi (AE-1475; ATTO Corporation Taito-ku, Tokyo, Japan). The following antibodies were used to detect the proteins:

Anti-CCNDBP1 (ab220275, Abcam, Cambridge, UK) at 1:2000 dilution; anti-EZH2 antibody (ab186006, Abcam) at 1:1000 dilution; anti-ATM antibody (ab78, Abcam) at 1:2000 dilution; anti-phospho S1981 ATM antibody (ab36810, Abcam) at 1:1000 dilution, anti-Chk2 antibody (No. 2662, Cell Signaling Technology, Inc., Danvers, MA, USA) at 1:1000 dilution; anti-phospho Chk2 (Thr68) antibody (No. 2661, Cell Signaling Technology, Inc., Danvers, MA, USA) at 1:1000 dilution; anti-Chk1 antibody (ab47574, Abcam) at 1:1000 dilution; anti-phospho Chk1 (S345) antibody (ab58567, Abcam) at 1:1000 dilution; anti-ATR antibody (ab2905, Abcam) at 1:10,000 dilution; anti-phospho ATR (T1989) antibody (ab227851, Abcam) at 1:3000 dilution; anti-Cdc25C antibody (ab226958, Abcam) at 1:1000 dilution; anti-CyclinD1 antibody (ab134175, Abcam) at 1:10,000 dilution; anti-p53 antibody (ab131442, Abcam) at 1:1000 dilution; anti-p21 antibody (ab188224, Abcam) at 1:1000 dilution; antimouse immunoglobulin G horseradish peroxidase (NA931-1ML; GE Healthcare Life Sciences, Pittsburgh, PA, USA); and antirabbit immunoglobulin G horseradish peroxidase (NA934-1ML; GE Healthcare Life Sciences, Pittsburgh, PA, USA). Protein bands were visualized using EzWestLumi plus (WSE-7120L; ATTO Corporation, Taito-ku, Tokyo, Japan) and LuminoGraphI (WSE-6100; ATTO Corporation, Taito-ku, Tokyo, Japan).

### 2.7. Histological Analysis

Tissues of thymus, spleen, and liver were collected at appropriate time points after the procedures and fixed in 10% formalin before embedding in paraffin for hematoxylin and eosin and immunohistochemical staining. Immunohistochemical staining was conducted with an anti-ATM antibody (ab78, Abcam, Cambridge, UK) at 1:1000 dilution and an anti-phospho S1981 ATM antibody (ab36810, Abcam, Cambridge, UK) at 1:250 dilution using the Vectastain Elite ABC mouse IgG kit (PK-6102, Vector Laboratories, Burlingame, CA, USA); an anti-CHK2 antibody (No. 2662, Cell Signaling Technology, Inc., Danvers, MA, USA) at 1:200 dilution; an anti-phospho Thr68 CHK2 antibody (No. 2661, Cell Signaling Technology, Inc., Danvers, MA, USA) at 1:100 dilution; an anti-ATR antibody (ab222820, Abcam, Cambridge, UK) at 1:100 dilution; an anti-phospho T1989 ATR antibody (ab223258, Abcam, Cambridge, UK) at 1:100 dilution; an anti-CHK1 antibody (ab47574, Abcam, Cambridge, UK) at 1:250 dilution; an anti-phospho S345 Chk1 antibody (ab47318, Abcam, Cambridge, UK) at 1:100 dilution; and an anti-KMT6/EZH2 antibody (ab191080, Abcam, Cambridge, UK) at 1:250 dilution using the Vectastain Elite ABC rabbit IgG kit (PK-6101, Vector Laboratories, Burlingame, CA, USA) and 3,3′-diaminobenzidine chromogen tablets (Muto Pure Chemicals, Tokyo, Japan). Thereafter, images from each tissue section were captured randomly, and quantitative analysis was performed with ImageJ software (version 1.6.0_20; National Institutes of Health, Bethesda, MD, USA) with an RGB-based protocol, as reported previously [28].

### 2.8. Microarray and Bioinformatic Analyses

The SurePrint G3 Human Gene Expression (v2) Microarray Kit (Agilent Technologies, Inc., Santa Clara, CA, USA) and SurePrint G3 Mouse Gene Expression (v2) Microarray Kit (Agilent Technologies, Inc., Santa Clara, CA, USA) GeneSpring GX, version 14.5.1 (Agilent Technologies, Inc., Santa Clara, CA, USA), were used to compare the gene expression levels in mock-transfected cells and in hepatocytes from *CCNDBP1*-transfected HLE and *Ccndbp1* knockout mice and wild mice. There were 6597 of the 26,083 genes in human and 7530 of the 27,122 genes in mice that were clustered hierarchically according to the level of gene expression, with more than two-fold differences in expression. The gene ontology terms were selected on the basis of Fisher’s exact test, followed by the Benjamini–Yekutieli correction method. The expressions in the genes were compared among the groups; genes with more than fivefold differences in expression were shown in the heat map.

### 2.9. Statistical Analyses

The obtained data were analyzed with either one-way or two-way factor repeated measures analysis of variance, followed by Bonferroni’s multiple comparison test or Student’s t-test using GraphPad Prism7 software (version 8.4.3; GraphPad, San Diego, CA, USA). A *p* value of ≤ 0.05 was considered to indicate statistical significance.

## 3. Results

### 3.1. Effect of CCNDBP1 Expression in HCC Cells on X-ray Irradiation

**Figure 1:**
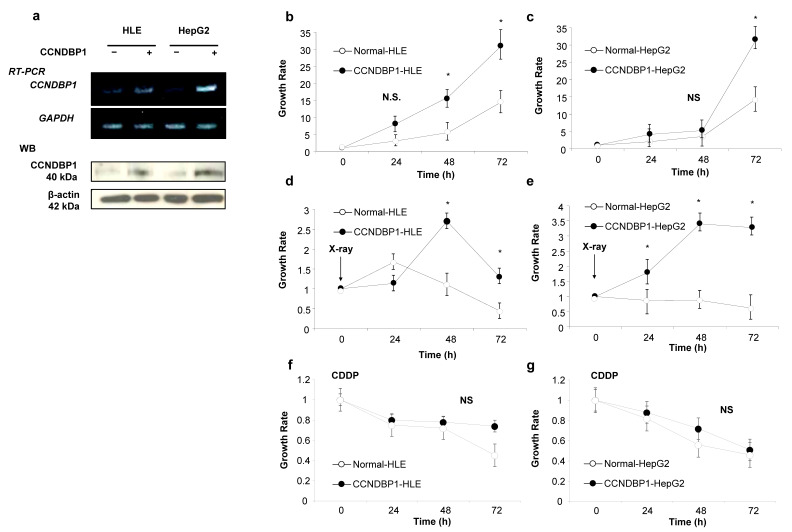
Effect of CCNDBP1 expression on HCC cell growth. The development of CCNDBP1-overexpressing cell lines and the cell growth of hepatocellular carcinoma (HCC) cell lines and the permanent clones overexpressing CCNDBP1 were determined by 3-(4,5-Dimethylthiazol-2-yl)-2,5-diphenyltetrazolium bromide (MTT) assay. (**a**) Representative reverse transcription polymerase chain reaction (RT-PCR) of CCNDBP1 and glyceraldehyde 3 phosphate dehydrogenase (GAPDH) and Western blotting (WB) of CCNDBP1 and β-actin are shown. (**b**,**c**) Growth of cell lines in normal culture condition with 10% fetal bovine serum. (**d**,**e**) Cell growth after irradiation with 0.8 Gy of X-ray. (**f**,**g**) Cell growth with 20 µM of CDDP. The values represent mean ± standard deviation (*n* = 3 for each group at the time points). * *p* < 0.05 and no statistical significance (NS) on two-way analysis of variance followed by Bonferroni’s multiple comparison test.

To examine the molecular function of CCNDBP1 on liver cancer cells, we produced *CCNDBP1*-overexpressing cell lines by transfecting plasmid DNA-expressing human *CCNDBP1* into cancer cell lines of HLE and HepG2. Figure 1a shows the *CCNDBP1* gene and CCNDBP1 protein expression in the transfected HLE and HepG2 cell lines. A slight endogenous level of CCNDBP1 expression can be seen in the cells, whereas significant overexpression of CCNDBP1 was achieved by the transfection. Compared with the mock-transfected cells, the *CCNDBP1*-overexpressing cells showed increased growth ratio in the culture condition containing 10% FCS, as determined by the MTT assay (Figure 1b,c). After irradiation, the CCNDBP1-expressing cell lines showed significantly higher growth rate than did the mock-transfected cells, in which cell growth was inhibited by X-ray (Figure 1d,e); this difference was not seen when treated with 20 µM of CDDP (Figure 1f,g). These results indicated that *CCNDBP1* overexpression in HCC stimulated cell growth under 10% FCS and resistance to X-ray.

### 3.2. Gene Expression Analyses in CCNDBP1-Overexpressed Cells and Ccndbp1 Knockout Mice

**Figure 2:**
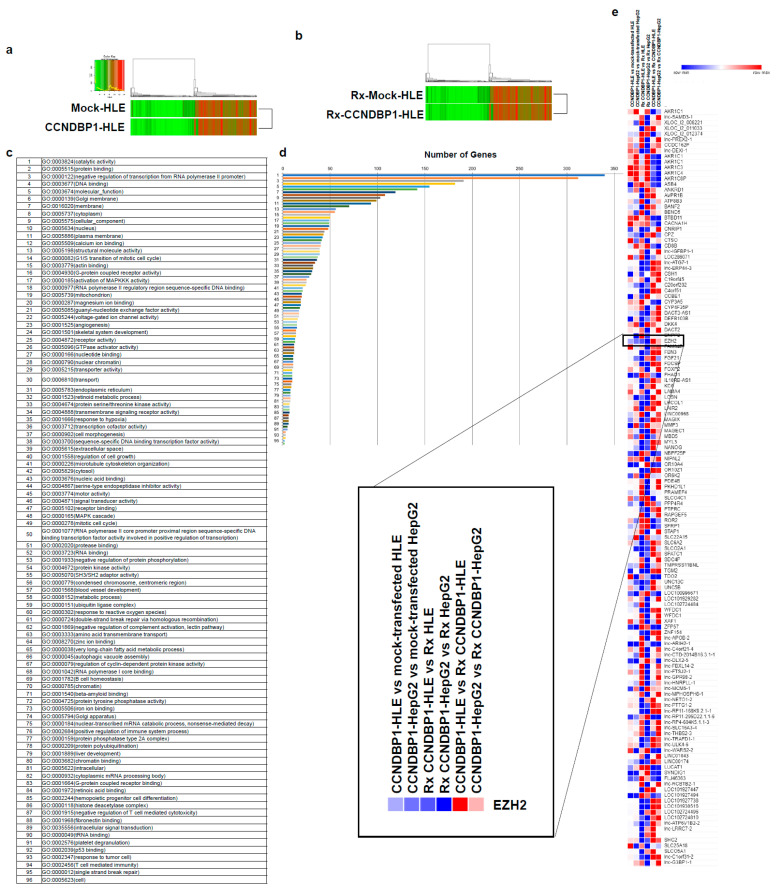
Microarray analyses in vitro. (**a**) Microarray analyses were performed to compare the levels of gene expression in mock-transfected HLE and CCNDBP1-transfected HLE. (**b**) X-ray irradiated mock-transfected HLE and X-ray irradiated CCNDBP1-transfected HLE. A total of 6597 genes with more than two-fold differences in expression were clustered hierarchically according to the level of gene expression. The color represents the expression level of the gene; green represents low degree of change in expression, whereas red represents high degree of change of expression. The color key and histogram are shown. (**c**) The gene ontology terms related with the genes that have more than two-fold differences between the mock-transfected HLE and CCNDBP1-transfected HLE are shown. (**d**) The number of genes included in each ontology are shown. (**e**) The expression level of genes showed more than two-fold changes in all comparisons between CCNDBP1-HLE and mock-transfected HLE, CCNDBP1-HepG2 and mock-transfected HepG2, Rx CCNDBP1-HLE and Rx mock-transfected HLE, Rx CCNDBP1-HepG2 and Rx mock-transfected HepG2, CCNDBP1-HLE and Rx CCNDBP1-HLE, and CCNDBP1-HepG2 and Rx CCNDBP1-HepG2. Rx, X-ray irradiation.

**Figure 3:**
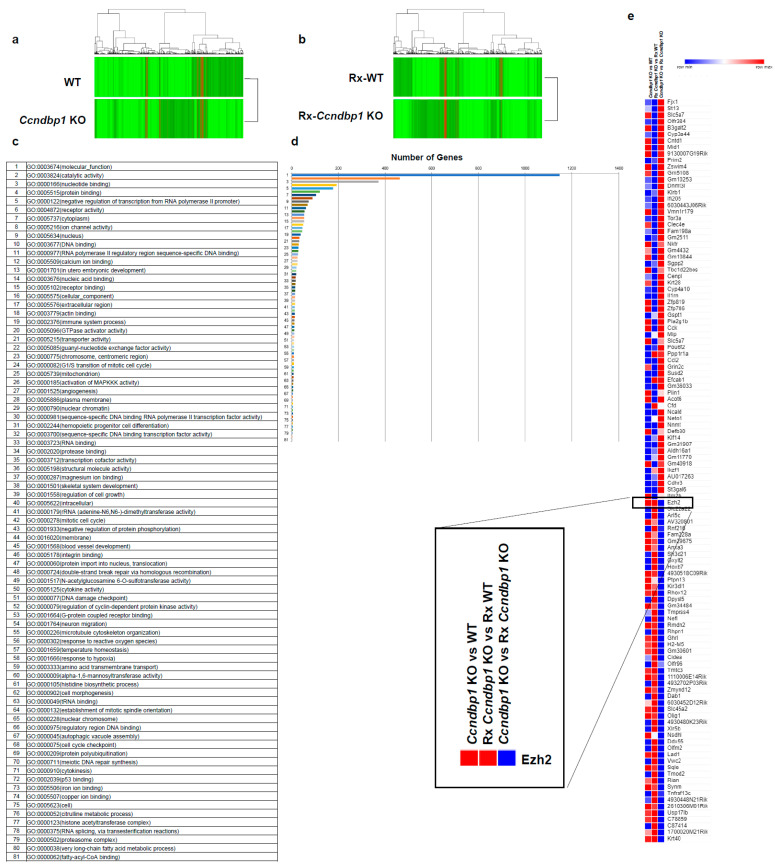
Microarray analyses in vivo. (**a**) Microarray analyses were performed to compare the levels of gene expression in WT and *Ccndbp1* KO mice. (**b**) X-ray irradiated WT and *Ccndbp1* KO mice. A total of 7530 genes with more than two-fold differences in the expression were clustered hierarchically according to level of gene expression. The color represents the expression level of the gene; green represents low degree of change in expression, whereas red represents high degree of change of expression. The color key and histogram are shown. (**c**) The gene ontology terms related with the genes that have more than two-fold differences between the WT and Ccndbp1 KO mice are shown. (**d**) The number of genes included in each ontology is shown. (**e**) The expression level of genes showed more than two-fold changes in all comparisons between *Ccndbp1* KO and WT, Rx *Ccndbp1* KO and Rx WT, and *Ccndbp1* KO and Rx *Ccndbp1* KO. WT, wild type; KO, knockout; Rx, X-ray irradiation.

To determine the molecular mechanism of *CCNDBP1*, the gene expression in mock-transfected and CCNDBP1-transfected HLE (Figure 2) and *Ccndbp1* knockout mice and wild type mice (Figure 3) were compared using DNA microarray analyses. The analysis of gene ontology terms after the hierarchical clustering of genes showed gene differences in terms of molecular function, catalytic activity, nucleotide, DNA, binding, and negative regulation if transcribed from RNA polymerase II promoter, RNA polymerase II regulatory region sequence-specific DNA binding, and nuclear chromatin in both cell and mice (Figure 2c,d and Figure 3c,d). Among the genes involved in these terms and those that showed more than two-fold difference in all comparisons of cells and mice with X-ray irradiation, we focused on the enhancer of zeste homolog 2 (EZH2), which showed expression differences both in vitro and in vivo (Figure 2e and Figure 3e). *EZH2* is an epigenetic silencer of the polycomb repressor complex 2 [29,30]; a negative regulator of DNA damage-related proteins of ATM [31] involved in the X-ray-induced DNA damage; and has been associated with the prognosis of several cancer entities [29,30,31]. The *EZH2* gene expression was lower in the *CCNDBP1*-overexpressed cell lines than in the mock-transfected cell lines and was lower in the irradiated *CCNDBP1*-overexpressed cell lines than in the nonirradiated *CCNDBP1*-overexpressed cells. These results implied that *CCNDBP1* expression and irradiation inhibited EZH2 expression. These findings were confirmed in the in vivo gene expression analysis. Figure 3e shows higher *Ezh2* in the *Ccndbp1* knockout mice under normal housing and after X-ray irradiation than in the wild type mice. Irradiation did not decrease the Ezh2 expression in the *Ccndbp1* knockout mice (Figure 3e). Overall, these results indicated that *Ccndbp1* controlled the expression of *Ezh2* in an inhibitory manner and that irradiation decreased *Ezh2* expression in a *Ccndbp1*-dependent manner.

### 3.3. CCNDBP1 Expression and DNA Damage-Related Proteins

**Figure 4:**
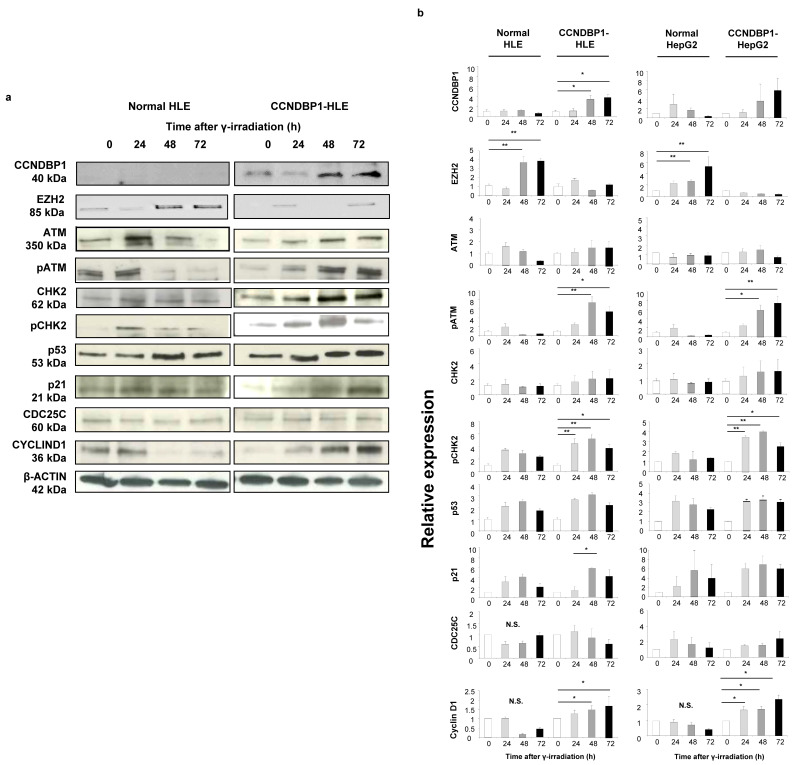
Effect of CCNDBP1 on the changes in various proteins in HCC cells after X-ray irradiation. (**a**) Western blotting of proteins related with the ATM–CHK2 pathway in the cells harvested at the indicated times after X-ray irradiation (immediately and at 24, 48, and 72 h). (**b**) The relative expression ratios of the proteins are shown. The values represent mean ± standard deviations (*n* = 5), * *p* < 0.05, ** *p* < 0.01 on one-way analysis of variance followed by Bonferroni’s multiple comparison test. CCNDBP1, cyclin d1 binding protein 1; EZH2, enhancer of zeste homolog 2; ATM, ataxia telangiectasia mutated; pATM, phosphorylated ataxia telangiectasia mutated; CHK2, checkpoint kinase 2; pCHK2, phosphorylated checkpoint kinase 2; CDC25C, cell division cycle 25 homolog C.

Based on the results obtained from the cell growth assay and microarray analyses of the cell lines, we examined the DNA damage-related proteins ATM and CHK2, which are involved in X-ray-induced DNA damage. Figure 4a shows the representative results of the Western blotting of the HLE cell lines, and Figure 2b summarizes the results of protein expression in the HLE and HepG2 cell lines. The expression of CCNDBP1 was activated by irradiation in a time-dependent manner, and it inhibited the expression of EZH2 protein, which is a negative regulator of ATM [31]. The X-ray irradiation induced the phosphorylation of ATM protein by inhibiting EZH2 expression after 24 h (Figure 4). In addition, overexpression of CCNDBP1 showed continuous inhibition of EZH2, followed by sustained phosphorylation of ATM for at least for 72 h after irradiation. Following the activation of ATM, CHK2 was continuously activated by its phosphorylation in the CCNDBP1-overexpressed cells. In addition, the higher increase in p53 and p21, inhibition of cdc25C, and gradual increase of cyclin D1 expression in the CCNDBP1-expressed cells than in the mock-transfected cells indicated cell cycle arrest upon irradiation and recovery of the tumor growth (Figure 4). Based on these results obtained in the in vitro assay, we next examined the molecular mechanisms of *Ccndbp1* in vivo using *Ccndbp1* knockout mice.

### 3.4. Effect of Ccndbp1 In Vivo

**Figure 5:**
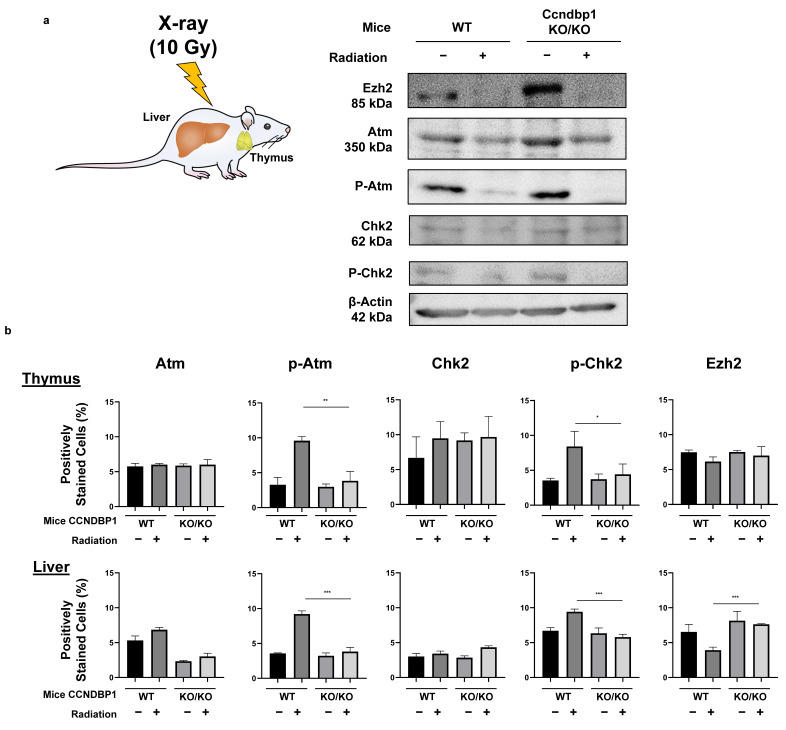
Effect of Ccndbp1 on the changes in various proteins in X-ray irradiated mice. (**a**) Western blotting of proteins related with the ATM–CHK2 pathway in mice thymic tissue harvested 12 h after X-ray irradiation. (**b**) Quantitative analyses of the immunohistochemical analyses of proteins in the thymus and liver of wild (WT) and Ccndbp1 knockout (KO) mice with or without X-ray irradiation. Representative images and a quantitative analysis of positively stained area are shown. The scale bar represents 100 µm. The values represent mean ± standard deviations (*n* = 5–6), * *p* < 0.05, ** *p* < 0.01, *** *p* < 0.001 on one-way analysis of variance followed by Bonferroni’s multiple comparison test.

The effect of *Ccndbp1* gene in mice was examined using the tissues collected from wild and *Ccndbp1* knockout mice before and after X-ray irradiation. Figure 5a shows the representative results of the Western blotting of thymus tissue. After irradiation, the expression levels of the Atm and Chk2 proteins were not different in both mice, but there was suppressed phosphorylation of the Atm and Chk2 proteins in the thymus cells, which are mostly lymphocytes, in the knockout mice, compared with that in the wild type mice (Figure 5a). In addition, the expression of Ezh2 was higher in the knockout mice than in the wild type mice (Figure 5a).

These results were confirmed with immunohistochemical staining of thymic and hepatic tissues. Figure 5b summarizes the results of the analysis of the protein expressions in the thymus and liver based on immunohistochemical staining (representative images are shown in Figure S1). The hepatocytes showed similar findings, with more clear differences and maintained expression of Ezh2 when irradiated (Figure 5b). The thymic tissues showed a relatively mild difference in Ezh2 expression. These results suggested that *Ccndbp1* activated the Atm–Chk2 pathway through inhibition of *Ezh2* expression. Therefore, knockout of *Ccndbp1* caused Ezh2 activation, which led to insufficiency of the Atm–Chk2 pathway and suppression of Chk2 phosphorylation upon irradiation in the knockout mice.

## 4. Discussion

In this study, we aimed to assess the molecular mechanism of CCNDBP1 and focused on the recovery from DNA damage. Indeed, our results demonstrated that *CCNDBP1* overexpression in HCC contributed to higher cell growth and resistance to X-ray-induced DNA damage and that this mechanism was dependent on the activation of the ATM–CHK2 pathway in cancer cells. These results were supported by the fact that abnormal activation of the ATM–CHK2 pathway had been associated with resistance to chemoradiotherapy and poor prognosis in nasal extranodal NK/T cell lymphoma [32]. Moreover, our results demonstrated that CCNDBP1-overexpressed cell lines have gained resistance to X-ray irradiation but not to cisplatin. This difference was partly explained by Ziegler et al., who reported that ionizing radiation caused the formation of numerous DSB and triggered a substantial activation of ATM–CHK2 signaling [33]; however, the DNA crosslinking agents of cisplatin triggered a substantial blockage of transcription, which was not reflected by an appreciable number of DSB and did not increase the expression of the ATM–CHK2 pathway [33]. In addition, we demonstrated that the suppressive effect of CCNDBP1 on EZH2, which is a histone H3K27 methyltransferase that was reported to be a negative regulator of ATM [31], contributed to the activation of the ATM–CHK2 pathway. This result was supported by a previous report showing that decreased EZH2 expression increased ATM phosphorylation and induced resistance to anticancer treatment [30]. These results were further supported by our analyses, which showed weaker activation of the ATM–CHK2 pathway after X-ray irradiation on the liver, spleen, and thymus in Ccndbp1 knockout mice. Recently, it has been reported that the expression of *CCNDBP1* gene could be regulated by DNA methylation [34]. This mechanism might be related with the modification of *CCNDBP1* expression in normal cells to gain malignant potential in the early stage of malignant transformation [10] and in cancer cells to be chemoresistant [25].

The limitations of our study included the lack of molecular-based analysis of the direct link between CCNDBP1 and EZH2 and of human HCC samples to enable application of the results to radiotherapy and chemotherapy resistance. In one report on progressive prostate cancer despite standard treatments, high response to chemotherapy was seen when the DNA repair system was inhibited by Olaparib, which is a poly adenosine diphosphate–ribose polymerase [35]. Similarly, *CCNDBP1* can be a therapeutic target in HCC with poor response to conventional therapy. Therefore, further basic research that will focus on the molecular mechanisms of *CCNDBP1* and *EZH2*, carcinogenic assay using *Ccndbp1* knockout mice with DNA damaging agents, and analyses of human samples will further bring important information. In addition, while we have utilized the male mice in this study to reduce the possible physiological stress caused by the menstrual cycles, future studies comparing the different functions of *Ccndbp1* based on sex may further reveal its mechanism.

## 5. Conclusions

According to our results, CCNDBP1 contributed to the activation of the ATM–CHK2 pathway by inhibiting EZH2 to ameliorate DNA damage. Therefore, management of CCNDBP1 expression in the tumor may recover the sensitivity to anticancer therapy.

## Supplementary Materials

The following are available online at https://www.mdpi.com/article/10.3390/jcm11030851/s1, Figure S1: Representative images of the immunohistochemical staining of the ATM–CHK2 pathway-related proteins in the thymus and liver of wild (WT) and Ccndbp1 knockout (KO) mice with or without irradiation of X-ray.
