# Supplementary material for: Cyclin D1 Binding Protein 1 Responds to DNA Damage through the ATM–CHK2 Pathway
Source: J Clin Med. 2022 Feb 6;11(3):851. doi: 10.3390/jcm11030851 (PMC8836734; doi:10.3390/jcm11030851)
Supplement: Supplementary file 1 [file jcm-11-00851-s001.zip › jcm-1567429 supplementary.pdf]

Supplementary Materials

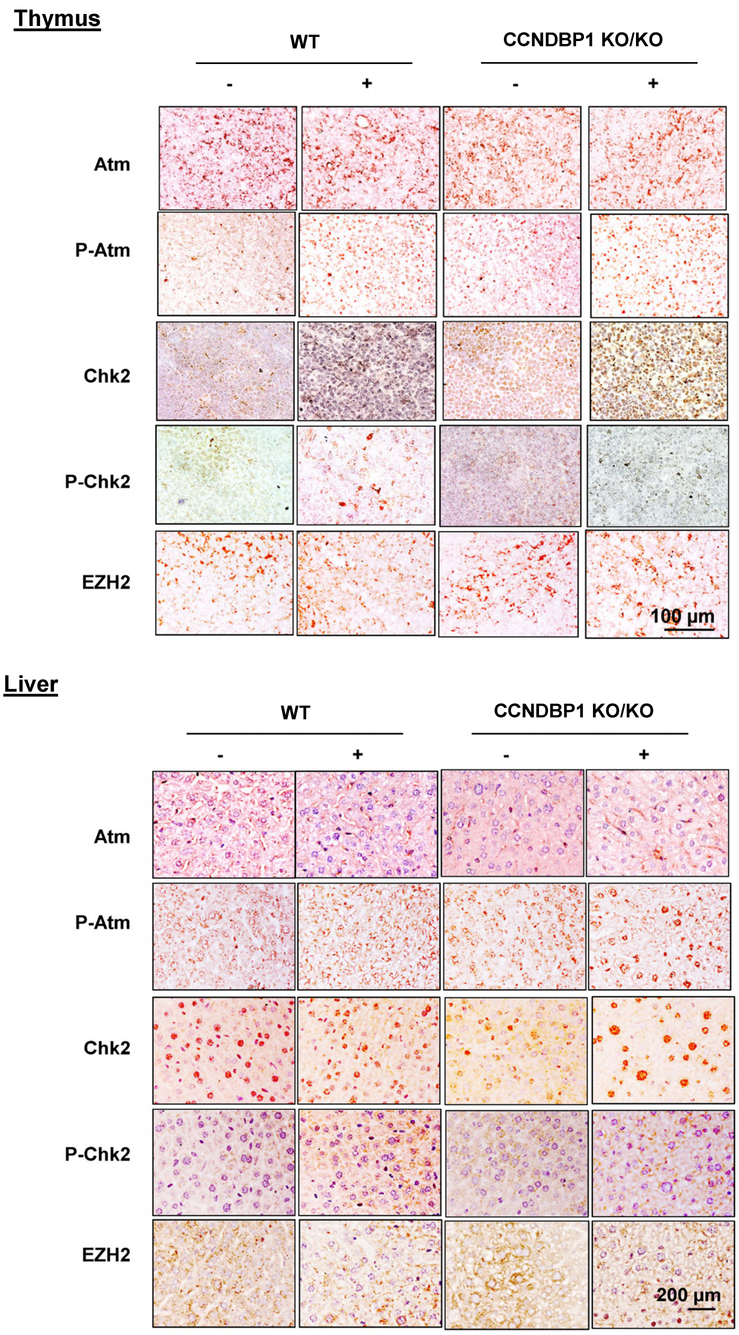

Figure S1: Representative images of the immunohistochemical staining of the ATM–CHK2 pathway-related proteins in the thymus and liver of wild (WT) and Ccndbp1 knockout (KO) mice with or without irradiation of X-ray.
